# Ammonia in the crosshairs: microbial targets for metabolic dysfunction–associated steatohepatitis prevention

**DOI:** 10.1172/JCI207921

**Published:** 2026-07-01

**Authors:** Vanessa A. Leone, Arion Kennedy

**Affiliations:** 1 Department of Animal & Dairy Sciences, University of Wisconsin-Madison, Madison, Wisconsin, USA.; 2 Department of Molecular and Structural Biochemistry, College of Agriculture and Life Sciences, North Carolina State University, Raleigh, North Carolina, USA.

## Abstract

Metabolic dysfunction–associated steatohepatitis (MASH) is increasingly linked to disruptions of the gut/liver axis, yet the microbial mechanisms driving disease progression remain incompletely defined. Here, Qu et al. have identified ileal microbial ammonia production by *Clostridium perfringens* as a mechanistic driver of epithelial barrier dysfunction and hepatic CD8^+^ T cell remodeling in MASH. In nonhuman primate and mouse models of MASH, the authors demonstrated that the glycine-based tripeptide DT-109 restored gut barrier integrity and attenuated FosB-mediated CCL5 expression in CD8^+^ T cells via inhibition of bacterial nitrite reductase A–mediated microbial ammonia production. These findings position microbial nitrogen metabolism as a tractable therapeutic target and highlight metabolite-focused microbiome interventions as a potential MASH intervention.

## Microbial ammonia in MASH

Metabolic dysfunction–associated steatotic liver disease (MASLD; formerly referred to as nonalcoholic fatty liver disease, NAFLD) is the leading cause of chronic liver disease worldwide, affecting an estimated 38% of the global population (1). This global prevalence continues to rise, with projections suggesting that up to 55% of individuals may be affected by 2040 (1). MASLD can progress to its more severe inflammatory form, metabolic dysfunction–associated steatohepatitis (MASH), in which fibrosis is strongly associated with increased risk of liver-related mortality (2).

Despite recent therapeutic advances, current pharmacological agents address only a fraction of the clinical needs in MASH. Resmetirom, a thyroid hormone receptor-β agonist, achieves histologic fibrosis improvement in approximately 25% of patients (3), while semaglutide, a glucagon-like peptide 1 receptor agonist, resolves MASH in roughly 60% of patients, with approximately 37% exhibiting improved fibrosis (4). Thus, a substantial proportion of patients remain without effective disease-modifying therapies, underscoring the need for additional mechanistically distinct interventions.

In this issue of the *JCI*, Qu et al. identified a mechanistically distinct microbial contribution to MASH pathogenesis, linking ileal ammonia production to epithelial barrier dysfunction and hepatic immune remodeling (5). Using complementary murine and nonhuman primate models of diet-induced MASH, the authors demonstrated that directly targeting microbial ammonia production, specifically by *Clostridium perfringens*, was sufficient to restore gut barrier integrity, reprogram CD8^+^ T cell responses, and improve MASH outcomes.

The gut/liver axis has emerged as a central driver of MASLD/MASH pathogenesis (6). Large-scale studies consistently demonstrate compositional shifts in the fecal microbiome of individuals with MASLD and MASH compared with healthy controls, including reductions in taxa such as *Bifidobacterium*, *Coprococcus*, and *Faecalibacterium prausnitzii* alongside enrichment of *Blautia*, *Dorea*, *Escherichia coli*, and *Klebsiella pneumoniae* (7). However, these signatures are highly heterogeneous, influenced by diet, varying comorbidities, race/ethnicity, and stage of liver fibrosis. Moreover, reliance on fecal microbiome profiling provides an incomplete view of host-microbe interactions, as it does not fully capture both the composition and functional activity of microbes residing in the small intestine (8). This distinction may be particularly important in MASLD/MASH, where the ileum serves as a key site for nutrient uptake, bile acid resorption, immune surveillance, and metabolite exchange within the gut/liver axis (8). These limitations underscore a central challenge in the field: microbial taxonomy alone and a reliance on stool have not yielded a unifying mechanistic explanation for disease progression.

Instead, increasing evidence suggests functional microbial outputs represent a critical link between microbial imbalances and disease pathophysiology. Among these outputs, ammonia is increasingly recognized as a key mediator of disease progression (9). Under normal physiological conditions, the liver efficiently detoxifies ammonia via the urea cycle; however, this capacity is impaired in MASLD and MASH, contributing to hyperammonemia (9). In parallel, the intestinal microbiome represents an additional source of ammonia, mediated, in part, by bacterial deamination of nitrogenous substrates, such as amino acids, and urease-dependent hydrolysis of urea (10). The intestinal microbiome’s contribution to ammonia production may be particularly relevant in the ileum, where regional microbial activity and proximity to portal circulation position this region of the small intestine as a key interface for metabolite exchange within the gut/liver axis (8). Dysbiosis-driven increases in ileal ammonia production, coupled with impaired barrier integrity, may enhance flux of this metabolite to the liver through portal circulation, exacerbating hepatocellular stress and metabolic dysfunction. Within this framework, specific ammonia-producing taxa, including *C*. *perfringens* (11), emerge as mechanistically plausible contributors to disease progression.

## Barrier integrity and CD8^+^ T cell polarization in MASH

**Figure 1:**
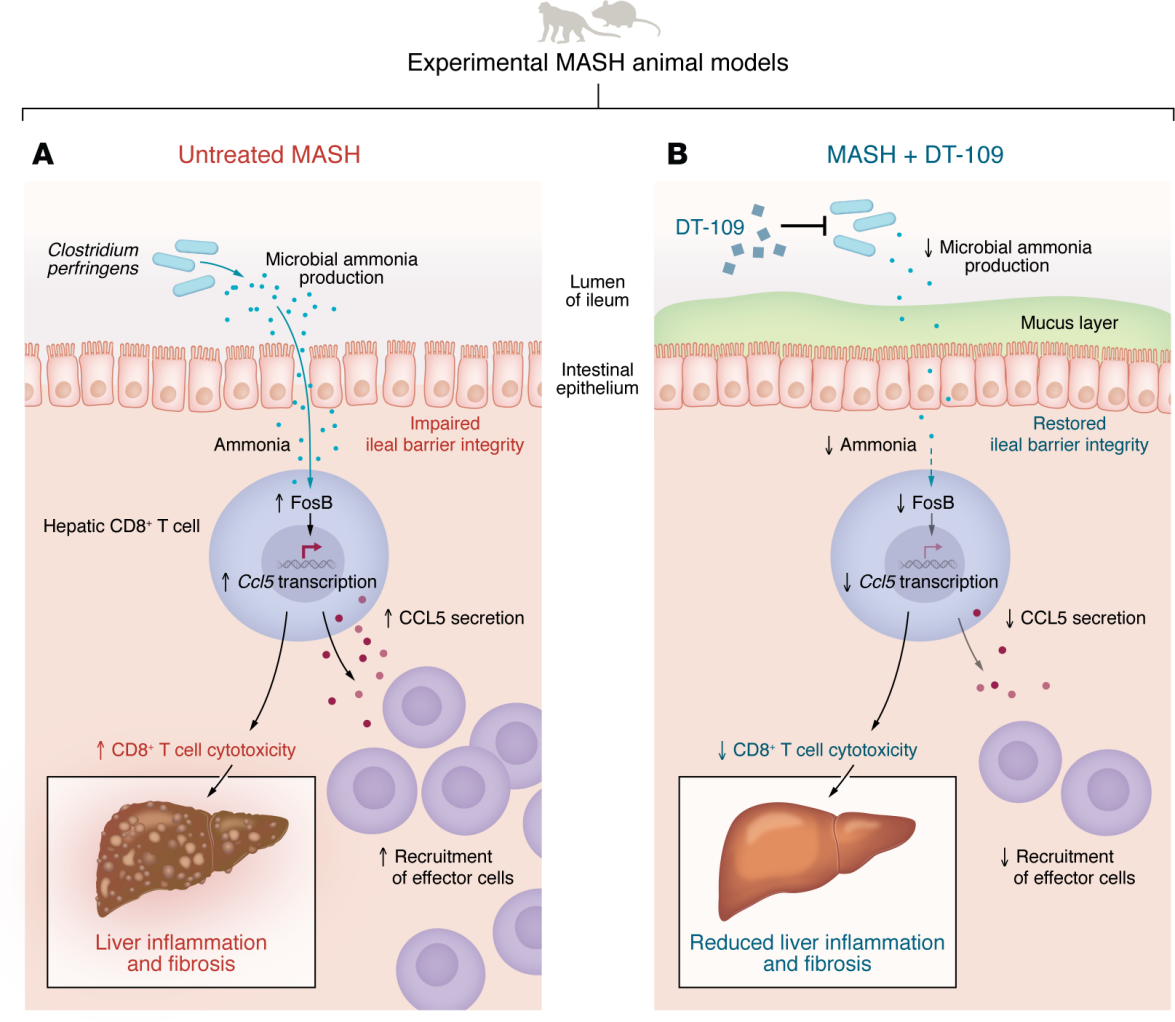
DT-109 inhibits microbial ammonia-driven CD8^+^ T cell activation in experimental MASH. (**A**) Qu et al. showed that ammonia production by nitrite reductase A (*NirA*)–expressing *C*. *perfringens* disrupts gut barrier function in diet-induced MASH models, which leads to increased exposure of hepatic CD8^+^ T cells to ammonia. Increased ammonia levels drive CCL5 expression mediated through FosB in hepatic CD8^+^ T cells, enhancing recruitment and cytotoxicity of effector cells. This resulted in severe liver inflammation and fibrosis. (**B**) In experimental MASH animal models, treatment with DT-109, a glycine-based tripeptide, inhibited NirA-mediated ammonia production in *C*. *perfringens*, resulting in downregulation of FosB-mediated CCL5 expression and reduced CD8^+^ T cell activation, effectively reducing liver injury and fibrosis (5).

A key advance of the study by Qu et al. is the identification of a spatially resolved mechanistic axis linking microbial ammonia production in the ileum to epithelial cell dysfunction and hepatic immune remodeling (Figure 1). Elevated ammonia has previously been shown to impair epithelial cell integrity through mitochondrial depolarization, oxidative stress, and inflammatory signaling via NF-κB, thereby promoting cytokine production and cellular injury (12). In animal models, elevated ammonia is further associated with loss of tight junction proteins, goblet cell depletion, mucus disruption, and recruitment of inflammatory cells that amplify tissue damage (13, 14).

Consistent with these observations, Qu et al. further demonstrated that microbial ammonia production by *C*. *perfringens* disrupted ileal barrier integrity, reducing expression of the tight junction protein occludin and perturbing the mucus layer by reducing mucin 2 protein expression in experimental MASH. Barrier dysfunction may facilitate microbial translocation, cytokine and damage-associated molecular pattern production, and antigen localization to regional lymph nodes, thereby promoting inflammatory immune activation.

Hepatic CD8^+^ T cells are established drivers of liver inflammation, immune cell infiltration, and fibrosis in MASH (15). Under MASH conditions, hepatic CD8^+^ T cell activation is shaped by cytokine signaling such as IL-15, antigen presentation, microbial metabolites such as acetate, and the gut microbiota (15, 16). Using heat-inactivated and nitrite reductase A (*NirA*–deficient) *C*. *perfringens*, Qu et al. demonstrated that bacterial ammonia production promoted upregulation of the chemokine CCL5 in hepatic CD8^+^ T cells, enhancing T cell cytotoxicity in experimental MASH. Mechanistically, ammonia directly induced FosB-mediated CCL5 expression in CD8^+^ T cells in vitro, linking this microbial metabolite directly to pathogenic hepatic CD8^+^ T cell polarization. Together with its effects on ileal barrier integrity, these findings position ammonia as a central mediator of gut-immune communication in MASH.

Beyond its effects on immunity through disruptions in barrier integrity, ammonia may also directly alter immune cell metabolism. Ammonia is detoxified primarily through glutamine synthetase–mediated conversion to glutamine, which can serve as a carbon and nitrogen donor or be hydrolyzed back to glutamate and ammonia by glutaminase (9). In immune cells, altered glutamine flux influences mTOR signaling, redox balance, and glycolytic programming (17). Elevated ammonia has been shown to induce lysosomal damage, mitochondrial dysfunction, and ROS production in CD8^+^ T cells, resulting in impaired metabolic fitness and reduced effector responses (18). In human CD8^+^ T cells, ammonia exposure reduces proliferation, IFN-γ and TNF-α production, degranulation, and antigen-specific cytotoxic responses (18). In contrast, Qu et al.’s findings suggest ammonia can also promote pathogenic CD8^+^ T cell activation in MASH, enhancing effector recruitment and cytotoxicity. Together, these results point to an important unresolved question regarding the extent to which ammonia directly reprograms hepatic resident CD8^+^ T cell subsets versus indirectly modulating peripheral CD8^+^ T cells that traffic to the liver. Qu et al. provided initial insight through single-cell RNA-sequencing analysis, demonstrating that DT-109 treatment altered transcriptional programs within hepatic T cell subsets in experimental MASH. Understanding these bidirectional interactions between barrier integrity, cytokine signaling, and local metabolites will be essential for the development of MASLD/MASH therapies that restore barrier function while reprogramming CD8^+^ T cell responses toward protective, rather than pathogenic, states.

## Inhibiting microbial ammonia production in MASH: proof of principle

DT-109 is a dual-action therapeutic that regulates cardiovascular disease, liver metabolism, and glycemic control. Previous preclinical studies demonstrated that DT-109 improved metabolic dysfunction in models of liver diseases, reduced hepatic steatosis, restored redox balance, and altered amino acid and nitrogen metabolism (19, 20). These effects were mechanistically linked to shifts in microbial bile acid metabolism that lowered hepatic exposure to toxic secondary bile acids (19). Thus, DT-109 likely acts through multiple complementary metabolic mechanisms across multiple host and microbial pathways. However, the precise mechanisms underlying DT-109’s therapeutic actions remain unclear. Qu et al. have now demonstrated that DT-109 reshaped the microbial community in experimental diet-induced MASH and inhibited ammonia production by *C*. *perfringens*. This reduction in microbial ammonia corresponded with restoration of ileal barrier integrity and attenuation of pathological CD8^+^ T cell polarization.

These findings move beyond correlative microbiome associations and provide functional validation for microbially produced ammonia as a therapeutic target in MASH. More broadly, the study supports the concept that targeting a single microbial function may be sufficient to reshape host responses and disease progression in certain conditions. Whether DT-109 treatment can fully reverse fully established MASH, however, remains an important open question.

## Microbiome-derived metabolites as targeted therapies in MASH

Our understanding of microbial contributions to disease pathogenesis is rapidly progressing as we go from considering taxonomy-based associations toward function- and metabolite-based targeting. The work by Qu et al. represents an important advance by demonstrating how precision modulation of microbial metabolism via a small molecule inhibitor can selectively “edit” microbial outputs to restore gut/liver homeostasis, overcoming limitations of approaches that use engineered bacteria to reduce intestinal ammonia burden and avoiding broader elimination of specific microbial taxa (reviewed in ref. 7). However, while *C*. *perfringens* produces ammonia, it has not been widely associated in human MASH cohorts as a central player in advanced disease. Whether DT-109 targets NirA-mediated ammonia production in other ammonia-producing bacterial species, such as *K*. *pneumoniae*, which has previously been associated with MASH (7), requires further investigation. Nonetheless, by reemphasizing microbially derived ammonia as a tractable mediator linking the ileal microbiome to hepatic immune dysfunction, Qu et al. provide a compelling framework for the development of small molecule inhibitors as metabolite-targeted therapies in MASH.

## Conflict of interest

The authors have declared that no conflict of interest exists.

## Funding support

This work is the result of NIH funding, in whole or in part, and is subject to the NIH Public Access Policy. Through acceptance of this federal funding, the NIH has been given a right to make the work publicly available in PubMed Central.

- VAL by a Gilead Sciences Research Scholars Award in Liver Disease and Accelerator funding from the Wisconsin Alumni Research Foundation.
- NIH funding awarded to AK (P30 DK034987).

## Version 1 07/01/2026

Electronic publication
